# Medical Education in Spain

**Published:** 1869-04-01

**Authors:** 


### FOREIGN ITEMS.

***Medical Education in Spain.***

The provisional government of Spain has decreed *absolute liberty of education.* Any Spaniard may found educational establishments, and open public courses of instruction of any kind whatsoever, without being obliged to submit the programme of leçons, to the minister, or to any representative of authority.

This innovation in established usage appears to be viewed differently by the two most prominent representatives of the medical press in Spain, *El Genio Medico-Quirurgico* and *El Siglo Medico*, which, by their antithetical attitudes, to quote the accomplished Redacteur-en-chef of the *Gazette Médicale* of Paris, "suggests the two philosophers of antiquity, Democritus and Heraclitus; the first-named journal recognizing in the new order of affairs only cause for joy, while the second groans over the inconsiderate enthusiasm of its cotemporary."

In Italy the attention of the profession is, just now, especially directed to the subject of malaria, of which there are such numerous sources beyond the Alps.

The *Gazette Médicale* of Turin expresses much astonishment that the rice fields should be permitted to be maintained. The danger to human health and life arising from the culture of rice has been indicated from time immemorial by hygienists and philanthropists; the medical press, special publications, sanitary and civil councils, medical and scientific societies and associations have, always and everywhere, insisted upon the pernicious effects of the emanations from inundated rice-fields. The peasants who labor, during a portion of the year, in these localities, by their cachectic appearance, their etiolated complexions, the engorgement of their abdominal organs, and the brevity of their lives, demonstrate most eloquently the deadly influences to which they are subjected.

The same authority estimates that the culture of sixteen hectolitres (3,200 lbs.) of rice costs a human life, and demands that the government shall disregard the pecuniary interests of proprietors, and suppress, by law, a branch of industry in which public health is sacrificed to private gain. It calls upon Italy to imitate the example of Portugal, which has prohibited rice-culture, to the great benefit of public health.—*Gazette Médicale, Paris.*

The *Gazette Médicale* of Venice calls the attention of the authorities to the dishonest and criminal practices of confectioners in adulterating their wares. Its remarks are suggested by the poisoning of a family consisting of a lady, child and servant, by eating nougat, which was proved by chemical analysis to have been colored with arsenite of copper (Scheeles green). The same coloring matter was detected in other articles in the same shop. The municipal authorities caused a placard designating the offence to be affixed to the establishment in question, and at the same time prohibited, under heavy penalties, the sale of any goods, manufactured at the establishment, already in the market.—*Gazette Médicale, Paris.*

NOTE.—It seems useless to warn our own citizens against the deletereous character of the confectionery so largely consumed throughout the West. If any one will take one of the common motto-wafers or kisses, and attempt to dissolve it in a glass of clear water, he will have an occular demonstration, more convincing than a chemical analysis, of their utter unfitness for food.

*Les Annales de Médicine* of Florence reports four cases of tænia originating from the use of raw meat, and quotes M. Weisse (of St. Petersburg), who, amongst the first to use raw meat in the treatment of the diarrhœa of infants, observed that many of his little patients contracted tænia solium; and this result follows as well the use of beef as of pork. The frequency of tænia amongst Abysinnians is attributed, by nearly every traveler who has written of that country, to the custom of eating raw meat, by the natives.

The translator has recently expelled a tænia solium from a patient, in this city, who had eaten no pork, but freely of raw beef. W. H.

The writer further suggests the importance of observing carefully the effects of fish diet, in originating entozoa, in countries where it constitutes the staple article of food, attributing to trout and the salmon the origin of the bothriocephalus.—*Ibid.*

